# Multifractal Cascade Modeling Reveals Fundamental Limits of Current Neuroimaging Strategies

**DOI:** 10.3390/s25237388

**Published:** 2025-12-04

**Authors:** Madhur Mangalam

**Affiliations:** Department of Biomechanics, University of Nebraska at Omaha, Omaha, NE 68182, USA; mmangalam@unomaha.edu

**Keywords:** multifractal cascade, brain dynamics, neuroimaging sampling, hierarchical sampling, fractal dimension, scale-invariance

## Abstract

Neuroimaging assumes spatial and temporal uniformity, yet brain activity exhibits a multifractal cascade structure with intermittent bursts and long-range dependencies. We use controlled simulations to test how well standard sampling strategies (random, grid-based, hierarchical; N = 10–2000 sensors) recover statistical properties—mean, variability, burstiness, and fractal dimension—from synthetic multifractal brain fields. Estimation errors deviate substantially from the classical $N^{-1/2}$ scaling expected under independent sampling. For higher-order statistics like burstiness, error reduction is remarkably flat in log–log space: orders-of-magnitude increases in sensor density yield virtually no improvement. Grid sampling performs best for fractal dimension at high densities; hierarchical sampling is more stable for burstiness. These results indicate that current neuroimaging fundamentally underestimates brain complexity and variability, with major implications for interpreting both healthy and pathological brain function.

## 1. Introduction

A hippocampal sharp-wave ripple lasts 50 ms and spans roughly 1 mm^3^, yet fMRI samples every 2 s across 27 mm^3^ voxels. This mismatch reflects a deeper problem: Neuroimaging instruments (EEG: 32–128 electrodes; MEG: 300+ sensors; fMRI: millimeter voxels sampled at 1–2 s intervals) assume spatial and temporal uniformity that the brain does not possess [1,2]. Growing evidence from animal models and humans points instead to multifractal cascade structure [3,4,5,6,7,8,9], where variability is distributed unevenly across scales, producing intermittent bursts (hippocampal sharp-wave ripples, cortical up-states) nested within ongoing fluctuations [10,11]. Scale-free avalanche behavior in human MEG [12] and cross-frequency phase synchronization [13] reveal dependencies that linear models cannot explain. If this multifractal structure is fundamental, then current sampling must systematically mischaracterize brain function.

Multifractal cascades violate the independent and identically distributed (IID) assumptions underlying neuroimaging [14,15,16,17,18,19,20]. Their hallmarks—scale invariance, long-range dependence, and regionally heterogeneous scaling [21,22,23]—mean that sampling limitations can underestimate variability, miss rare but consequential events, and obscure hierarchical organization [24,25,26,27]. Standard preprocessing of brain activity may suppress informative variability, plausibly contributing to under-detection of complexity changes in neurological and psychiatric conditions [28,29,30,31]. Classical intuitions about sampling efficiency ($N^{-1/2}$ convergence) become unreliable [32], with implications for both normal function and pathology.

Most prior work has examined temporal multifractality using long time series (EEG, MEG, fMRI) [3,4,5] and methods such as multifractal detrended fluctuation analysis [33,34,35]. We address a complementary but distinct issue: the spatial multifractality of brain activity fields, i.e., how variability is distributed across cortical regions at a given moment. This spatial focus interrogates fundamental measurement constraints: how many sensors are needed, which layouts are robust, and whether estimation error follows classical $N^{-1/2}$ convergence or the slower rates expected in multifractal systems.

We use controlled simulations to probe what different sampling methods can recover from synthetic brain fields generated by multifractal cascades. We examine (i) how sampling density (from N = 10 to N = 2000) affects estimation accuracy for mean, variability, burstiness, and fractal dimension; (ii) how random, grid-based, and hierarchical layouts compare across regions with varying complexity; and (iii) whether estimation error follows the IID benchmark $N^{-1/2}$ [32] or exhibits slower convergence characteristic of multifractal cascade structure [14,15,16,17,18,19,20].

Brain dynamics span roughly ten orders of magnitude in space and time [36,37,38,39], making sampling limits particularly acute. By comparing estimates to known ground truth, we identify which statistical measures degrade, which sampling strategies remain resilient, and where sensor density offers diminishing returns. The results have direct implications for neuroimaging design: recognizing multifractality motivates sampling and inference strategies matched to hierarchical dependencies across spatial and temporal scales, potentially improving sensitivity to clinically relevant changes [40,41,42,43] and bridging cellular and large-scale dynamics [36,37,38,39].

## 2. Methods

### 2.1. Generation of Synthetic Brain Data

#### 2.1.1. Lattice and Global Settings

All simulations were performed in Matlab 2024a (MathWorks, Natick, MA, USA) on a 256 × 256 lattice. We ran 100 independent simulations and simulated T = 20 time steps per simulation. Sample sizes were as follows: N ∈ {10,50,100,500,1000,2000,4000,10,000}.

#### 2.1.2. Template Generation (Mask and Regions)

We constructed a disc-like brain mask $B\;\subseteq \;{\left\{1,\ldots ,256\right\}}^{2}$ centered at the image midpoint with a radius 0.4 of the image size. The binary disc was randomly eroded by retaining only pixels where an auxiliary uniform noise field satisfied U < 0.9, then Gaussian-smoothed (σ = 2 pixels) and thresholded at 0.5. Mesoscale heterogeneity was imposed by six Gaussian “blob” regions inside B. For each blob i ∈ {1,…,6}, a random in-mask center was selected; a Gaussian field with width 256/8 was formed, multiplied by B, and thresholded at 0.3 to assign its voxels to a region label *i*. Voxels in B not assigned by any blob were set to a catch-all region, yielding K = 7 disjoint regions ${\left\{R_{r}\right\}}_{r=1}^{7}$ that partition B.

#### 2.1.3. Regional Multiplicative Cascade

We initialized F ≡ 1 on B and generated a quad-tree multiplicative cascade independently within each $R_{r}$. The recursion depth was D = 8. At each level, the current sub-quadrant received an independent lognormal multiplier:

$$M=\exp\;\left(\sigma_{r}W\;-\;0.5\sigma_{r}^{2}\right),\;W∼N\left(0,\;1\right),$$

with $\sigma_{r}\;=\;B_{r}/\sqrt{12}$. Region-specific burst factors were drawn once per region per simulation as follows:

$$B_{r}=B\left(0.8+0.4u_{r}\right),\;u_{r}∼\mathrm{Unif}\left(0,\;1\right),\;B=0.6.$$

Multipliers were applied only within $R_{r}$. No renormalization across levels was applied. Region fields were merged and masked to B to form $S_{1}$.

#### 2.1.4. Temporal Evolution

To induce temporal persistence without additional regional modulation, the field evolved according to the following:

$$S_{t}=\alpha S_{t-1}+\left(1-\alpha \right)C_{t}+\eta_{t},\;\alpha =0.8,\;t=2,\ldots ,20,$$

where each $C_{t}$ is a fresh cascade on B generated as above but using a uniform region mask (a single region); $\eta_{t}$ is IID Gaussian noise with standard deviation 0.05 supported on B. Non-negative activity was enforced at each step via $S_{t}\leftarrow \text{max}\left(S_{t},\;0\right)$; values outside B were zeroed.

#### 2.1.5. Multifractal Analysis

We estimated the spatial multifractal spectrum with the Chhabra–Jensen direct method [44,45]. For dyadic box sizes $\epsilon \;=\;2^{k}$ starting at 4 pixels and increasing up to $\epsilon_{\text{max}}\;=\;\lfloor \text{min}(\mathrm{rows},\mathrm{cols})/2\rfloor$, we tiled the field and computed normalized box masses $\mu_{k}\left(\epsilon \right)$ from the mean activity within each box. For moment orders q ∈ [−10, 10] in steps of 1, we formed $p_{k}\left(q,\epsilon \right)\;=\;\mu_{k}{\left(\epsilon \right)}^{q}/\sum_{j}\mu_{j}{\left(\epsilon \right)}^{q}$ and estimated

$$\alpha \left(q\right)=\frac{\sum_{k}p_{k}\left(q,\epsilon \right)\;\log\mu_{k}\left(\epsilon \right)}{\log\epsilon },\;f\left(\alpha \left(q\right)\right)=\frac{\sum_{k}p_{k}\left(q,\epsilon \right)\;\log p_{k}\left(q,\epsilon \right)}{\log\epsilon }$$

as slopes from linear regressions versus logϵ across the available scales. Only *q*-values whose fits satisfied |r| ≥ 0.95 (i.e., $R^{2}\;\geq \;0.90$) were retained. All analyses used the final snapshot $S_{20}$ within the mask B.

### 2.2. Sampling Strategies

We compared three strategies at each *N*: *Random* draws *N* distinct in-mask voxels uniformly; *Grid* uses a stride $\Delta \;=\text{max}\left(1,\lfloor \sqrt{|B|/N}\rfloor \right)$ and scans row–column order, accepting in-mask coordinates until exactly *N* are collected; *Hierarchical* allocates ⌊N/K⌋ samples per region and distributes the remainder uniformly at random over regions, then samples uniformly without replacement within each $R_{r}$.

### 2.3. Statistical Analysis

#### 2.3.1. Mean and Standard Deviation

Let I = {(i,j)∈B} and $V\;=\;\{S_{t}\left(i,j\right):\left(i,j\right)\in I,\;t\;=\;1,\ldots ,20\}$. Ground-truth mean and standard deviation SD were as follows:

$$\mu_{\mathrm{true}}=\frac{1}{\left|V\right|}\sum_{v\in V}v,\;\sigma_{\mathrm{true}}=\sqrt{\frac{1}{\left|V\right|}\sum_{v\in V}{\left(v-\mu_{\mathrm{true}}\right)}^{2}}.$$

For a sample $\Omega \;=\;{\left\{\left(i_{\ell },j_{\ell }\right)\right\}}_{\ell =1}^{N}$, $V_{\Omega }\;=\;\left\{S_{t}\left(i_{\ell },j_{\ell }\right):\ell \;=\;1,\ldots ,N;\;t\;=\;1,\ldots ,20\right\}$, and

$$\mu_{\mathrm{est}}=\frac{1}{|V_{\Omega }|}\sum_{v\in V_{\Omega }}v,\;\sigma_{\mathrm{est}}=\sqrt{\frac{1}{|V_{\Omega }|}\sum_{v\in V_{\Omega }}{\left(v-\mu_{\mathrm{est}}\right)}^{2}}.$$

#### 2.3.2. Burstiness

Burstiness combined pooled skewness and excess kurtosis:

$$B_{\mathrm{true}}=\left|{\mathrm{skew}}_{\mathrm{true}}\right|+\text{max}\left(0,\;{\mathrm{kurt}}_{\mathrm{true},\mathrm{ex}}\right),\;B_{\mathrm{est}}=\left|{\mathrm{skew}}_{\mathrm{est}}\right|+\text{max}\left(0,\;{\mathrm{kurt}}_{\mathrm{est},\mathrm{ex}}\right).$$

#### 2.3.3. Fractal Dimension

For ground truth, we used box counting on $S_{20}$ within B. Let $\mu_{20}$ and $\sigma_{20}$ be the spatial mean and standard deviation over I; we thresholded at $\tau \;=\;\mu_{20}\;+\;0.5\;\sigma_{20}$ to obtain a binary pattern and counted occupied boxes for ϵ ∈ {2,4,8,16,32,64}. We regressed logN(ϵ) on logϵ overall valid scales and took the negative slope as $D_{\mathrm{true}}$, clamped to [0, 2]; if fewer than three valid scales existed, $D_{\mathrm{true}}\;=\;\mathrm{NaN}$. For sampled data, we reconstructed a sparse image having observed values at the sampled coordinates and applied the same box-counting estimator when N ≥ 20; otherwise $D_{\mathrm{est}}\;=\;\mathrm{NaN}$.

#### 2.3.4. Error Metrics and Aggregation

For each statistic X ∈ {μ,σ,B,D}, sampling method, and *N*, percentage relative error was as follows:

$${\mathrm{Error}}_{\%}\left(X\right)=\left|\frac{X_{\mathrm{est}}-X_{\mathrm{true}}}{X_{\mathrm{true}}}\right|\times 100\%,$$

with ϵ-regularization applied to $X_{\mathrm{true}}$ for σ,B,D when needed. Errors were averaged across the 100 simulations.

## 3. Results

### 3.1. Visualization of Synthetic Cascade Fields

**Figure 1:**
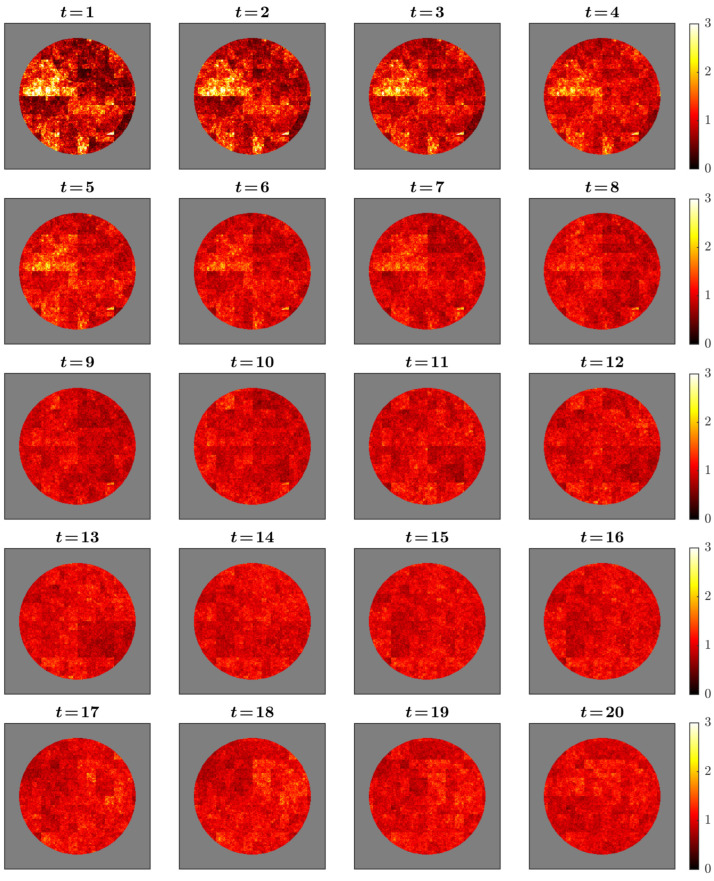
Temporal evolution of the simulated cascade field across 20 time points. Values are shown within a disc-shaped mask with a slightly irregular boundary using a “hot” colormap (darker = lower amplitude, brighter = higher). The sequence illustrates spatial intermittency and mild temporal persistence characteristic of multiplicative cascades. Regional heterogeneity arises solely from region-specific modulation of cascade multipliers. Panels are illustrative of model behavior and do not assert neurophysiological realism.

The simulated cascade fields captured the intended multifractal dynamics (Figure 1). Activity patterns appeared as intermittent clusters of high intensity embedded within broader regions of lower amplitude, consistent with the spatially heterogeneous distribution characteristic of multiplicative cascades. Successive time points showed mild temporal persistence, yielding gradual rather than abrupt transitions in spatial structure. Regional contrasts arose solely from controlled modulation of cascade multipliers across predefined masks, introduced to examine how spatial heterogeneity affects sampling accuracy. These patterns illustrate the internal behavior of the cascade model under the chosen parameters and do not assert correspondence to neurophysiological organization.

**Figure 2:**
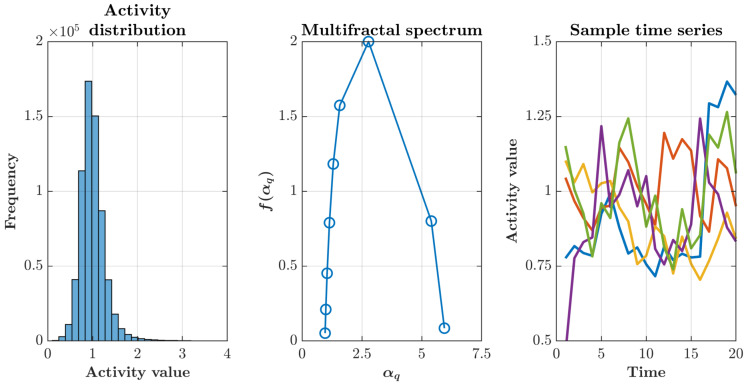
Statistical properties of the multifractal cascade simulation. (**Left**) Pooled histogram of activity values across all voxels and time points, showing a right–skewed, approximately lognormal distribution with a long upper tail—typical of multiplicative cascades. (**Middle**) Singularity (multifractal) spectrum $f(\alpha_{q})$ versus $\alpha_{q}$ obtained using the Chhabra–Jensen direct method [44,45]. Its broad, concave shape with a clear maximum indicates genuine multifractality; the spectrum’s width reflects intermittency and spatial heterogeneity. (**Right**) Sample time series from five randomly chosen locations illustrating heterogeneous temporal dynamics with intermittent bursts and mild persistence. Together, these features validate the cascade generator and motivate testing how different sampling layouts recover statistics under multifractal structure.

The statistical properties of this simulated activity confirmed its multifractal character. Activity values across all time points exhibited a right-skewed, approximately lognormal distribution characteristic of multiplicative cascade processes, with a concentration of low-to-moderate values and a heavy tail extending toward rare but intense activity peaks (Figure 2, left). Analysis of the mass exponent τ(q) revealed nonlinear scaling, providing rigorous confirmation of multifractality through deviation from the linear relationship expected for monofractal processes (Figure 2, middle). Time series extracted from five randomly selected points within the brain mask demonstrated heterogeneous temporal dynamics, with each location displaying distinct patterns of burstiness and autocorrelation while maintaining global statistical properties consistent with the underlying multifractal structure (Figure 2, right). Quantitative analysis of the multifractal spectrum confirmed robust spatial multifractality across all simulations. The spectrum width, defined as $\Delta \alpha \;=\;\alpha_{\text{max}}\;-\;\alpha_{\text{min}}$, averaged Δα = 4.88 ± 0.20 (mean ± SD,n = 100 simulations), indicating multifractality.

### 3.2. Sampling Performance Across Statistics

**Figure 3:**
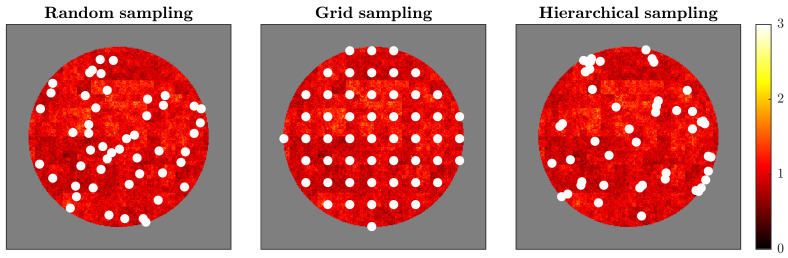
Comparison of sampling strategies applied to the multifractal brain simulation. The figure shows three sampling approaches (50 sampling points each, indicated by white dots) overlaid on the final time point of simulated brain activity. (**Left**) Random sampling distributes points uniformly across the brain mask without considering the underlying spatial structure. (**Middle**) Grid sampling arranges points in a regular geometric lattice, providing systematic spatial coverage. (**Right**) Hierarchical sampling allocates points proportionally across predefined regions, ensuring representation from all spatial zones while maintaining randomness within each region. White dots represent sensor locations. The background colormap shows activity intensity from low (dark red/black) to high (bright yellow). Each strategy reflects different principles for sensor placement, with implications for capturing multifractal dynamics: random sampling assumes spatial homogeneity, grid sampling prioritizes geometric regularity, and hierarchical sampling respects regional structure while balancing coverage.

**Figure 4:**
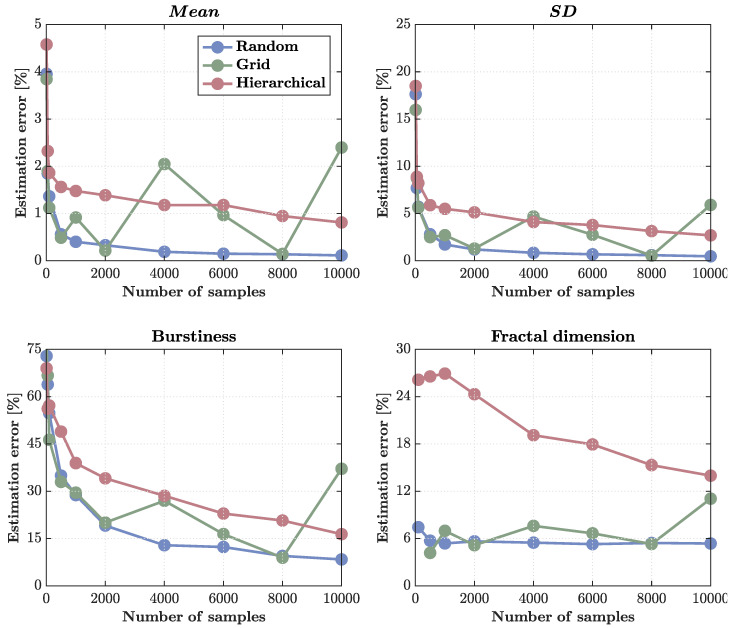
Estimation errors for different sampling methods across sample sizes. Percentage errors are shown for four statistics—mean, standard deviation (SD), burstiness, and fractal dimension—estimated from the multifractal cascade simulation as a function of sample size (10–10,000 samples). (**Top left**) Mean estimation error shows that only random sampling achieves classical convergence, reaching near-zero error (<0.1%) by $N\;∼\;10^{4}$. Grid sampling fluctuates with sample size, and hierarchical sampling fails to converge, maintaining residual errors around 1% due to redundancy within clusters. (**Top right**) SD errors show similar trends: random sampling converges rapidly, grid sampling remains noisy, and hierarchical sampling shows persistent bias and elevated error (3–5%) even at high *N*. (**Bottom left**) Burstiness estimation error remains high for all methods, reflecting the intrinsic difficulty of capturing intermittent, heavy-tailed dynamics. Random sampling performs best (∼10% at $N\;∼\;10^{4}$), while hierarchical and grid sampling converge slowly and irregularly. (**Bottom right**) Fractal dimension errors are smallest and most stable under random sampling (∼5%), with grid sampling moderately higher and hierarchical sampling persistently overestimating error (>15%) due to spatial clustering and reduced effective sample size. Overall, hierarchical sampling shows weak to no convergence for even first-order statistics due to the detrimental effect of spatial redundancy in clustered designs.

Estimation accuracy improved with increasing sample size for all metrics, but the rate and stability of convergence differed sharply across statistics and sampling strategies (Figure 3 and Figure 4).

First-order statistics (mean and standard deviation SD) were the most reliably estimated. For the mean, random sampling achieved near-perfect convergence, with errors dropping below 0.5% by N ∼ 200 and approaching zero (<0.1%) at $N\;∼\;10^{4}$. Grid sampling performed moderately well but showed irregular fluctuations with sample size, likely reflecting aliasing with the cascade’s underlying spatial periodicities. Hierarchical sampling failed to achieve full convergence: its errors plateaued around 1%, indicating that clustered, region-based allocation introduces redundancy that limits statistical independence even at large *N* (Figure 4, top left).

The same pattern held for the standard deviation (SD) (Figure 4, top right). Random sampling again achieved the lowest and most stable errors (<1% at high densities), grid sampling remained erratic, and hierarchical sampling retained a systematic upward bias, with residual errors of 3–5% at the highest *N*. This lack of convergence suggests that hierarchical clustering effectively reduces the number of independent observations, yielding an effective sample size smaller than the nominal *N*.

Higher-order statistics exhibited markedly poorer scaling. Burstiness estimation remained dominated by large errors across all methods (Figure 4, bottom left). Random sampling outperformed the others, reducing errors from roughly 70% at minimal sampling to about 10% at $N\;∼\;10^{4}$, whereas grid and hierarchical sampling converged slowly and irregularly, plateauing at ∼15% and ∼30%, respectively. These patterns reflect the intrinsic difficulty of recovering heavy-tailed, intermittent statistics from finite, spatially correlated samples.

Fractal dimension estimation displayed similar trends (Figure 4, bottom right). Random sampling again produced the most consistent results (∼5% error across the entire range), grid sampling fluctuated between 5–10%, and hierarchical sampling systematically overestimated errors (>15%), again reflecting the reduced independence of clustered observations.

Overall, these results demonstrate that random sampling offers the most statistically efficient and stable recovery of both first- and higher-order quantities, while hierarchical designs suffer from redundancy that prevents convergence even for simple moments. The findings emphasize that, in multifractal fields, the structure of sampling matters as much as the number of samples: clustered layouts degrade effective resolution and bias estimates, whereas decorrelated designs (random or jittered grids) preserve information across scales.

### 3.3. Scaling Properties

**Figure 5:**
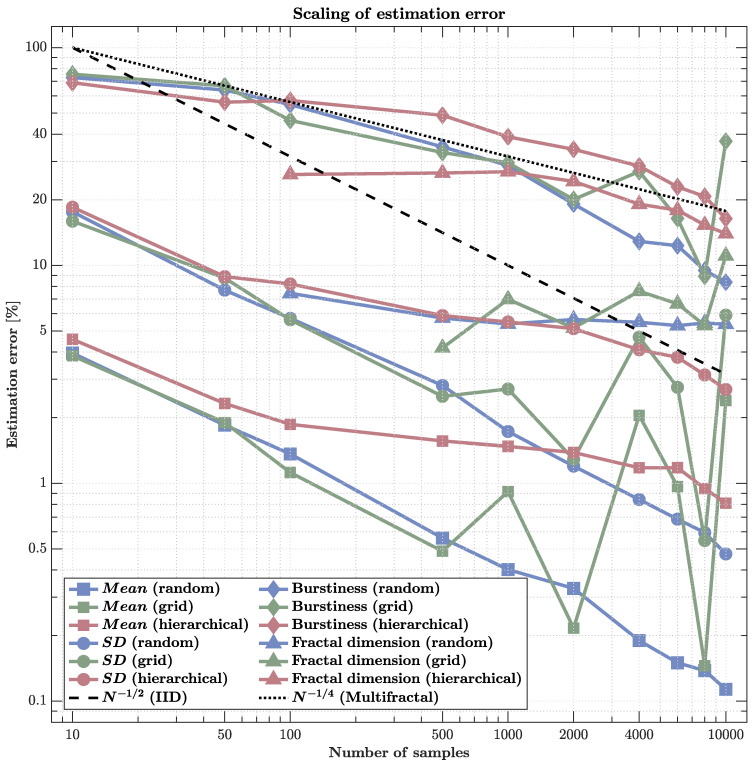
Scaling of estimation error across sampling schemes and metrics (log–log). Errors are shown for mean, standard deviation (SD), burstiness, and fractal dimension as sample size *N* increases. The black dashed line indicates the classical IID rate $N^{-1/2}$; the dotted line shows the slower $N^{-1/4}$ rate typical of multifractal structure. Mean and SD errors for random sampling closely track $N^{-1/2}$, while for grid sampling, they initially do so before flattening at high *N*. By contrast, under hierarchical sampling, even first-order statistics (mean and SD) do not converge: errors remain elevated and non-monotonic with *N*, indicating that the branching, clustered design yields highly redundant observations, reduces effective sample size, and can bias moment estimates. Higher-order quantities (burstiness and dimension) converge far more slowly for all schemes—roughly near $N^{-1/4}$—with errors still ≳5% at $N\;∼\;10^{4}$. Clearly, estimation accuracy depends as much on the sampling *structure* as on *N*. Hierarchical strategies are ill-suited for estimating moments; decorrelated designs (random or jittered grid) are preferable. Moreover, even large increases in *N* provide only modest gains for higher-order, multiscale statistics.

Analysis of the relationship between sample size and estimation error in log–log coordinates revealed qualitatively distinct scaling regimes across metrics and sampling strategies (Figure 5).

First-order statistics (mean and SD) showed scaling near the classical $N^{-1/2}$ rate under random sampling, achieving subpercent errors at high densities ($N\;∼\;10^{4}$). Grid sampling followed a similar trend but exhibited mild irregularities due to aliasing with a cascade structure. Hierarchical sampling, however, deviated sharply: its errors remained roughly constant or even fluctuated with *N*, indicating non-convergence and effective loss of independence among samples caused by clustering within correlated regions.

Higher-order metrics demonstrated fundamentally slower scaling. Burstiness errors decreased approximately as $N^{-1/4}$, consistent with multifractal cascade behavior, and remained above 5% even at maximal sampling. Fractal dimension estimates showed similar sluggish improvement, saturating around 10–15% error despite increasing *N* by two orders of magnitude. These scaling behaviors collectively indicate that statistical efficiency collapses in multifractal fields: the gains from additional sensors diminish sharply once spatial correlations dominate, leaving residual estimation bias that cannot be reduced by density alone.

These results have direct implications for neuroimaging and large-scale sensing. While simple averages converge efficiently, metrics reflecting intermittency and scale-dependent organization (e.g., burstiness, fractal dimension) display structural limits on sampling precision. Thus, denser sensor arrays may provide little benefit unless sensor placement actively decorrelates measurements or adapts to the cascade hierarchy itself.

## 4. Discussion

Our simulations reveal a fundamental constraint: if brain activity exhibits multifractal cascade properties, then current neuroimaging systematically underestimates its complexity. The non-classical scaling between sample size and estimation error ($N^{-1/4}$ rather than $N^{-1/2}$) indicates that even substantial increases in sensor density yield only modest gains for complex statistics. This directly challenges the assumption that incremental growth in sensor count proportionally improves measurement accuracy. Notably, even 2000 sampling points—far beyond routine practice—left large errors for burstiness and fractal dimension, raising doubts about whether standard neuroimaging can recover the full complexity of neural activity. These results point to a measurement challenge that transcends any single modality and argue for rethinking acquisition and analysis rather than simply scaling hardware.

The poor and often non-monotonic behavior of hierarchical sampling for the mean and SD is expected in clustered designs applied to multifractal fields. First, clustering induces positive intra-cluster correlation ρ, so the effective sample size collapses from *N* to $N_{\mathrm{eff}}\;\approx \;N/\left(1\;+\;(m\;-\;1)\rho \right)$, where *m* is the average within-region sample count and ρ is the intra-region correlation. In multifractal fields, ρ > 0 at many scales, making neighboring observations highly redundant and biasing moment estimates toward regional centroids. Second, our allocation rule (a floor of ⌊N/K⌋ per region) creates unequal inclusion probabilities that are not corrected by weights; small, low-variance regions become over-represented while intermittent, high-amplitude “hot spots” are under-sampled. This design effect depresses $N_{\mathrm{eff}}$ and explains why errors do not decrease with *N* as $N^{-1/2}$ and can even fluctuate when *N* crosses dyadic cascade scales (aliasing with the cascade hierarchy). Practically, this means that adding sensors within the same regional clusters yields diminishing returns for first-order statistics.

A second, practical implication is that sampling *design* matters. Hierarchical sampling outperformed random and grid-based approaches for higher-order statistics, lowering errors by approximately 15–30% while maintaining representation across regions. This suggests that sensor placement informed by anatomy and functional organization could improve accuracy without increasing sensor count. By contrast, regular grids—adequate for means—were least effective at capturing burstiness and fractal dimension, suggesting that uniform layouts systematically miss localized, intermittent structure. Taken together, these findings support biologically informed, multi-resolution arrays over technologically convenient uniform distributions.

The heavy-tailed activity distributions observed here also have implications for preprocessing. Standard filtering and averaging procedures designed to reduce noise may suppress precisely those bursts that carry information, thereby masking individual differences and transients of clinical or cognitive relevance. In parallel, the slow convergence of higher-order estimates suggests that classical pipelines may underestimate true variability, potentially encouraging overconfidence in group-level summaries [46,47].

In our simulations, apparent “regional” differences in estimation accuracy reflect methodological artifacts—interactions between scaling properties and spatial sampling schemes—not intrinsic biological variation. Certain combinations of scaling behavior may require denser sampling to yield stable multifractal estimates. We therefore refrain from drawing inferences about dynamic brain properties solely from simulations and view these results as guidance for designing and testing sampling strategies that must be validated with empirical data. This interpretation is consistent with persistent difficulties in characterizing some areas (e.g., subcortical structures) despite hardware advances [48,49]; however, any claim about true regional differences must await direct empirical confirmation. Region-specific underestimation of burstiness and complexity, if present in vivo, could be consequential for neurological and psychiatric disorders given the diagnostic and prognostic value of neural variability [28,29,30,31].

These limitations are not insurmountable. Several complementary approaches could substantially improve the recovery of multifractal structure from limited samples. Model-based reconstruction that encodes cascade-like priors (e.g., lognormal/multiplicative cascades with long-range dependence) together with spatial regularizers consistent with intermittency may recover more structure than purely geometric interpolation [50]. Multiscale integration across modalities can leverage complementary spatial and temporal sensitivities via joint inverse modeling, cross-modal constraints, and harmonized preprocessing [51,52,53]. Adaptive sampling that reallocates sensors toward emergent hotspots—using information-theoretic criteria [54] in a closed loop—may optimize coverage of intermittent events. Finally, analyses explicitly tailored to multifractal processes—for example, wavelet-leader/wavelet-based estimators and detrended fluctuation approaches [33,34,35]—augmented with robust/quantile estimators and surrogate-data tests, may extract more information from sparse or noisy data than standard statistics.

Several limitations warrant acknowledgment. The synthetic brain model simplifies anatomy and physiology; incorporating white-matter connectivity and region-specific dynamics is a clear next step. Parameter choices were theory-driven and should be validated against empirical neuroimaging. Although we focused on spatial sampling, temporal constraints also shape inference and interpretability. Moreover, realistic sensor noise and physiological artifacts were not modeled here and should be included in future work to increase ecological validity.

Our findings are predictive, not confirmatory: they arise from controlled simulations that assume multifractal cascade structure and idealized sampling conditions. We have not demonstrated that identical error-scaling deviations occur in empirical neuroimaging, nor that our cascade generator is a mechanistic model of neural activity. The simulations establish what would happen if multifractal structure is present; empirical validation requires multimodal datasets with harmonized preprocessing, validated complexity metrics, and cross-scale analyses. This represents a clear priority for future work.

Within these constraints, the results carry a clear implication: if neural activity exhibits multifractal cascade properties, commonly used sampling strategies will systematically under-represent emergent complex dynamics [55]. Progress will not come primarily from denser uniform arrays—which our results show provide diminishing returns—but from optimized sampling strategies, multiscale integration, and analysis methods explicitly designed for intermittent, nonlinear structure. Given the hierarchical organization of the brain—from neurons to networks to systems—measurement and analysis should be comparably hierarchical [56,57,58,59]. If multifractality proves fundamental to neural dynamics, acknowledging it will be pivotal for next-generation diagnostics and interventions that operate effectively in the presence of cascade-like variability. The question is no longer whether to account for multifractality, but how.

## Data Availability

Data sharing does not apply to this article as no new data were created or analyzed in this study.
